# Increasing Local Excitability of Brainstem Respiratory Nuclei Reveals a Distributed Network Underlying Respiratory Motor Pattern Formation

**DOI:** 10.3389/fphys.2019.00887

**Published:** 2019-07-23

**Authors:** Rishi R. Dhingra, Werner I. Furuya, Tara G. Bautista, Thomas E. Dick, Roberto F. Galán, Mathias Dutschmann

**Affiliations:** ^1^Division of Systems Neurophysiology, The Florey Institute of Neuroscience and Mental Health, Parkville, VIC, Australia; ^2^Division of Pulmonary, Critical Care and Sleep, Department of Medicine, Case Western Reserve University, Cleveland, OH, United States; ^3^Department of Electrical Engineering and Computer Science, Case Western Reserve University, Cleveland, OH, United States

**Keywords:** respiratory pattern formation, ataxic breathing, excitation-inhibition balance, respiratory microcircuit, synchronization, oscillator

## Abstract

The core circuit of the respiratory central pattern generator (rCPG) is located in the ventrolateral medulla, especially in the pre-Bötzinger complex (pre-BötC) and the neighboring Bötzinger complex (BötC). To test the hypothesis that this core circuit is embedded within an anatomically distributed pattern-generating network, we investigated whether local disinhibition of the nucleus tractus solitarius (NTS), the Kölliker-Fuse nuclei (KFn), or the midbrain periaqueductal gray area (PAG) can similarly affect the respiratory pattern compared to disinhibition of the pre-BötC/BötC core. In arterially-perfused brainstem preparations of rats, we recorded the three-phase respiratory pattern (inspiration, post-inspiration and late-expiration) from phrenic and vagal nerves before and after bilateral microinjections of the GABA(A)R antagonist bicuculline (50 nl, 10 mM). Local disinhibition of either NTS, pre-BötC/BötC, or KFn, but not PAG, triggered qualitatively similar disruptions of the respiratory pattern resulting in a highly significant increase in the variability of the respiratory cycle length, including inspiratory and expiratory phase durations. To quantitatively analyze these motor pattern perturbations, we measured the strength of phase synchronization between phrenic and vagal motor outputs. This analysis showed that local disinhibition of all brainstem target nuclei, but not the midbrain PAG, significantly decreased the strength of phase synchronization. The convergent perturbations of the respiratory pattern suggest that the rCPG expands rostrally and dorsally from the designated core but does not include higher mid-brain structures. Our data also suggest that excitation-inhibition balance of respiratory network synaptic interactions critically determines the network dynamics that underlie vital respiratory rhythm and pattern formation.

## Introduction

The concept of central pattern generator (CPG) networks that produce rhythmic physiologic motor activities emerged from studies concerned with invertebrate flight, swimming, feeding, and locomotion (Marder and Calabrese, 1996). The CPG concept is also applied for the generation of rhythmic vertebrate motor activities including locomotion (Grillner and Wallén, 1985; McCrea and Rybak, 2008; Kiehn, 2016), chewing (Yamada and Yamamura, 1996), swallowing (Jean, 2001), sniffing (Deschênes et al., 2012), and breathing (Richter, 1982; von Euler, 1983; Feldman, 1986). The concept of CPGs as a substrate of complex neural network dynamics was extended to the rhythmic neural activity of the cortex (Yuste et al., 2005). Thus, until today, the CPG concept is central to the understanding of neural circuits.

The respiratory CPG (rCPG) of vertebrates is the only motor network whose motor pattern is generated continuously throughout life to serve the vital function of oxygen uptake and carbon dioxide excretion. In contrast, all other motor CPG networks are episodic and require higher commands or sensory stimulation for their activation. The contemporary view on the structure and function of the rCPG implicates a respiratory rhythm generator called the pre-Bötzinger complex (pre-BötC; Smith et al., 1991; Feldman and Del Negro, 2006) that drives a pattern formation circuit located nearby within the Bötzinger complex (BötC). This anatomically localized core of the rCPG generates a sequential three-phase motor pattern of inspiration, post-inspiration and late-expiration (see Smith et al., 2009; Richter and Smith, 2014; Del Negro et al., 2018). Currently, several models for the generation of the three-phase respiratory motor rhythm and pattern are debated. A popular model depends on interconnected excitatory neurons with distinct bursting properties in the pre-BötC (see Rybak et al., 2014) that initiates inspiratory bursts. The rhythmogenic pre-BötC core also contains inspiratory GABAergic and glycinergic neurons that are thought to provide inhibition to expiratory neuron populations in the neighboring BötC and thereby inter-nuclear connectivity is thought to form an inhibitory ring of mutual inhibition between inspiratory, post-inspiratory and late-expiratory neurons (Richter, 1982; Rybak et al., 2004; Smith et al., 2007, 2009; Ausborn et al., 2018). This rhythm- and pattern-generating core is embedded into a larger anatomically functionally compartmentalized lateral respiratory column spanning from the caudal medulla to dorsal pons (Alheid et al., 2004; Rybak et al., 2004; Smith et al., 2007; Alheid and McCrimmon, 2008; Dutschmann and Dick, 2012). The respiratory areas outside of the core circuit, such as the pontine respiratory group (see Dutschmann and Dick, 2012) or the caudal ventral respiratory group of the medulla oblongata are thought to provide tonic modulatory inputs to the core or to relay the phasic respiratory activity to spinal (e.g., phrenic, intercostal, or abdominal motor neurons) or cranial (e.g., vagal-laryngeal, hypoglossal motor neurons) respiratory motor pools.

The recent discovery of an oscillator for post-inspiration, the so-called post-inspiratory complex (PiCo, Anderson et al., 2016), located rostro-medially to the pre-BötC/BötC respiratory core gave rise to an alternative model called the triple oscillator hypothesis (Ramirez and Baertsch, 2018). In this hypothesis, anatomically distinct independent oscillators for inspiration (pre-BötC), post-inspiration (PiCo), and late-expiration (RTN/pFRG) are mutually coupled via inhibitory and excitatory synaptic interactions to generate the three-phase motor pattern of respiration. While the triple oscillator hypothesis is an intriguing new concept for the generation of multi-patterned breathing activity, it requires further experimental and computational verification and validation. Thus, the common view of a layered CPG architecture (see McCrea and Rybak, 2008) that includes a separate rhythm generator (pre-BötC), which is connected to a downstream motor pattern formation network, currently prevails.

A recent publication from our laboratory tested the hypothesis of network degeneracy and redundancy by using *in situ* brainstem perfused brainstem preparations in which brainstem circuits were isolated by transection before re-perfusion at three distinct levels (caudal to the pre-BötC, just rostral to the pre-BötC or just rostral to the RTN/pFRG) (Jones and Dutschmann, 2016). The results confirmed the essential role of the pre-BötC for respiratory rhythm generation but failed to verify the anatomical location of the pattern formation circuit within the medulla alone. Specifically, preparations that maintained the anatomical integrity of all designated key nuclei for the generation of the respiratory rhythm and pattern, including the pre-BötC, BötC, RTN/pFRG, and PiCo, failed to produce a sequential three-phase motor pattern and instead produced a monophasic respiratory motor output that was synchronously expressed in functionally distinct respiratory motor nerves such as inspiratory phrenic, inspiratory/post-inspiratory vagal, and expiratory iliohypogastric nerves. We concluded that respiratory pattern formation requires essential ponto-medullary synaptic interactions to generate a physiologic three-phase respiratory motor pattern. Indeed, anatomically, the rCPG is classically defined as bilaterally organized neuronal columns stretching from the pontine Kölliker-Fuse nuclei through the caudal ventral respiratory group (Alheid et al., 2004; Rybak et al., 2004; Smith et al., 2007, 2009; Alheid and McCrimmon, 2008; Dutschmann and Dick, 2012). Therefore, ponto-medullary synaptic interactions within the rCPG are still considered to be the key mechanism underlying respiratory pattern formation.

As introduced above, the core circuit for respiratory rhythm and pattern generation is commonly anatomically localized to the pre-BötC and the neighboring Bötzinger complex BötC. In the present study, in addition to the pre-BötC/BötC core circuit, we investigated the role of the following nuclei in respiratory rhythm and pattern formation in the perfused brainstem preparation: (1) The nucleus of the solitary tract (NTS) is a major relay for peripheral respiratory sensory inputs (Kubin et al., 2006; Zoccal et al., 2014). In addition, our recent work indicates that the NTS might also be an integral part of the pattern formation network as a component of the dorsal respiratory group (DRG) (Bautista and Dutschmann, 2014b; Jones et al., 2016; Dhingra et al., 2017). (2) The pontine Kölliker-Fuse nuclei (KFn) is a critical area within the pontine respiratory group that controls the centrally mediated inspiratory off-switch (Cohen and Shaw, 2004; Ezure and Tanaka, 2006; Dutschmann and Dick, 2012) and gates post-inspiratory laryngeal adductor activity (Dutschmann and Herbert, 2006) and inspiratory hypoglossal nerve activity (Bautista and Dutschmann, 2014a) in the perfused brainstem preparation. (3) The periaqueductal gray is a midbrain area that relays and integrates limbic and cortical respiratory commands into brainstem respiratory circuits (Faull et al., 2019). In the perfused brainstem preparation, it was shown that chemical stimulation of PAG compartments triggered pronounced respiratory modulation, whereas pharmacological lesion of the PAG had no effect on the generation of the stationary respiratory motor pattern in the perfused brainstem preparation (Farmer et al., 2014).

To test the hypothesis that the pre-BötC/BötC core circuit is embedded within an anatomically distributed pattern generating network, we locally increased neuronal excitability by locally disinhibiting these anatomically and functionally distinct respiratory nuclei of the brainstem and midbrain via microinjection of the GABA(A) receptor antagonist bicuculline. We used the perfused brainstem preparation (Paton, 1996) for our experiments because it generates a three-phase respiratory motor pattern in the absence of sensory feedback (the lungs are removed; there are no respiratory movements; and there is no effect of carotid sinus nerve transection; see Farmer et al., 2016) and thereby allows for the investigation of the respiratory network’s CPG mechanisms. While local pharmacologic disinhibition will not reveal whether an area is active at baseline and likely activates nearby silent neurons, analysis of the effects of local disinhibition on the respiratory motor pattern offers a method to functionally assess the net connectivity of a given area with the whole of the respiratory network. Our data demonstrate that local disinhibition of any target nuclei, except the PAG, severely disrupts the respiratory motor pattern and significantly decreases the strength of synchronization between inspiratory and post-inspiratory motor outputs. Therefore, our data indicate that, contrary to contemporary models, the central generation of the breathing movements depends on synaptic interactions across a distributed neural circuit, rather than consisting of an anatomically localized core circuit.

## Materials and Methods

Experimental protocols were approved and conducted with strict adherence to the guidelines established by the Animal Ethics Committee of The Florey Institute of Neuroscience and Mental Health, Melbourne, Australia.

### Perfused Brainstem Preparation

Experiments were performed in juvenile Sprague-Dawley rats of either sex (*N* = 37 rats, 17–30 days post-natal) using the arterially perfused *in situ* brainstem-spinal cord preparation as described previously (Paton, 1996; Dutschmann et al., 2000; Dhingra et al., 2017). Briefly, rats were anesthetized by inhalation of isoflurane (2%) until they reached a surgical plane of anesthesia. The rats were then transected below the diaphragm and transferred to an ice-cold bath of artificial cerebrospinal fluid (aCSF, in mM: 125 NaCl, 3 KCl, 1.25 KH_2_PO_4_, 2.5 CaCl_2_, 1.25 MgSO_4_, 25 NaHCO_3_, 10 D-glucose) for pre-collicular decerebration. After decerebration, the lungs and heart were removed. Next, the descending aorta, phrenic, vagal, and hypoglossal nerves were dissected for subsequent cannulation or recording. Note that analysis of hypoglossal nerve activity was not included in the present study. However, an analysis of the effect of local disinhibition of brainstem target areas on the generation of the inspiratory motor pattern is presented in Dhingra et al. (2019). The preparation was then transferred into the recording chamber. The descending aorta was cannulated with a double-lumen catheter for perfusion and measurement of perfusion pressure. The preparation was perfused with aCSF containing sucrose (4.5 × 10^−3^ g/ml) for oncotic pressure, warmed to 31°C using a peristaltic pump, recirculating water bath and heat exchanger (ELMI, TW-2.02). The perfusion circuit also contained two bubble traps and a nylon filter (Millipore, 100 μm pore size) to prevent embolism. The perfusate was continually bubbled with carbogen (95% O_2_/5% CO_2_) to maintain constant chemosensory drive. Phrenic and vagal nerves were mounted in suction electrodes to measure respiratory motor output. Nerve potentials were amplified (10,000×, Warner Instruments, DP-311), filtered (0.01–10 kHz), digitized (AD Instruments, PowerLab 16/35), and stored on a computer using LabChart software (AD Instruments).

### Experimental Protocol

After the initial stabilization of the eupneic three-phase respiratory rhythm, we first recorded 10 min of the baseline respiratory motor pattern. In the present work, we targeted the NTS (*N* = 9 preparations), pre-BötC/BötC (*N* = 11 preparations), KFn (*N* = 10 preparations), and PAG (*N* = 7 preparations) as potential components of the respiratory pattern formation circuit. To functionally identify the target site coordinates, using a triple barreled pipette, we first mapped the target site with glutamate microinjections (50 nl, 10 mM). Glutamate microinjections within the BötC, NTS, and KFn evoked a bradypnea, whereas glutamate microinjections within the pre-BötC and PAG evoked a tachypnea. Once appropriate functionally identified injection site coordinates were identified, we microinjected bicuculline (50 nl, 10 mM), a GABA(A) receptor antagonist, to locally disinhibit and consequently locally increase the excitability of the target site. Next, we microinjected pontamine sky blue (50 nl, 2% w/v in aCSF) for *post hoc* histologic verification of injection site locations. This procedure was then repeated on the contralateral side. Note that after functional and histologic confirmation of injection site coordinates in an initial cohort, in subsequent experiments, we locally microinjected bicuculline and pontamine sky blue according to the established site coordinates without functional identification of target sites. After local disinhibition of the target site, we recorded 10 min of the respiratory motor pattern for measurement of respiratory phase durations, respiratory rhythm variability, and phase synchronization properties.

**Figure 1:**
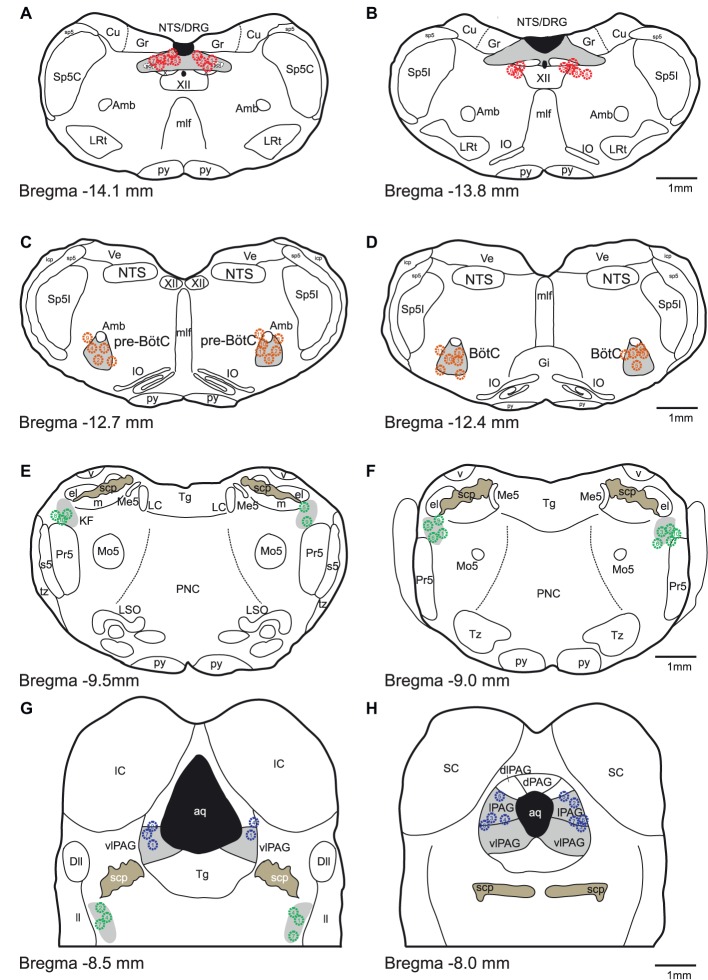
Histologic verification of injection sites. **(A-H)** Schematic drawings indicate the locations of bicuculline microinjections are presented from caudal to rostral. All microinjections were histologically identified within the designated target areas. Red, orange, green and blue symbols indicate NTS, pre-BötC/BötC, KFn and PAG injection sites, respectively.

At the conclusion of the experiment, brainstems were removed, post-fixed in paraformaldehyde (4% w/v in PBS), cryo-protected in sucrose (20% w/v in PBS), cryo-sectioned (50 μm thickness), and counter-stained with neutral red to verify microinjection sites (Figure 1).

### Data Analysis

Respiratory phase durations and respiratory rhythm variability were assessed from PNA. PNA was first high-pass filtered to remove DC fluctuations with a zero-phase FIR filter (300 Hz), and then integrated (100 ms time constant) and normalized to have zero mean and unit variance. Breaths were detected using a threshold-crossing algorithm to determine the onset and offset times of PNA. Events were manually inspected to ensure that no false-positive breaths were included in subsequent analyses. From these event times, we measured the mean respiratory period (*T*_TOT_), inspiratory (*T*_I_) and expiratory (*T*_E_) phase durations, and their coefficient of variation (CV). To qualitatively compare the respiratory rhythm variability evoked by local disinhibition of the target areas, we generated Poincaré plots of *T*_TOT_*(n)* versus *T*_TOT_*(n+1)* using the entire *T*_TOT_ time series. Points in such Poincaré plots provide information about the relationship between one respiratory period and its successor (Fishman et al., 2012).

### Motif Occurrence

To qualitatively assess the similarities and differences between local disinhibition of the different target sites, we scored the occurrence of eight representative motor pattern motifs during 3-min epochs recorded after bicuculline microinjection. To compare the relative frequencies of motif occurrence across experiments, we normalized the raw counts to the total number of motifs in each epoch, thereby yielding the probability of motif occurrence.

### Phase Synchronization Analysis

**Figure 2:**
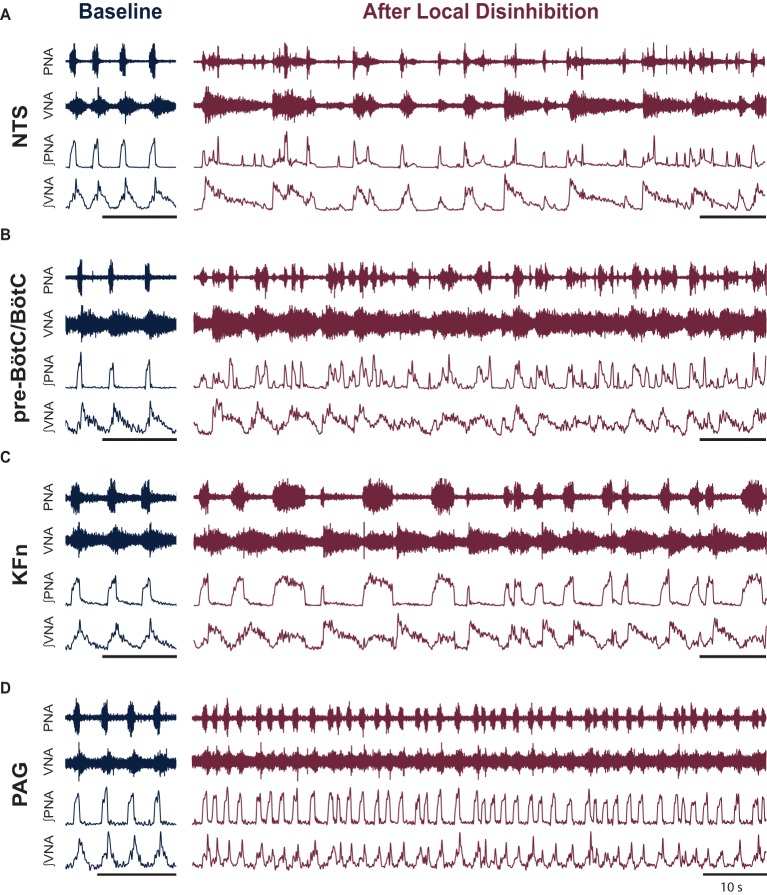
Local disinhibition of anatomically distributed brainstem-, but not midbrain-, respiratory areas disrupts the eupneic three-phase respiratory motor pattern. To test the hypothesis that the pre-BötC/BötC core circuit is embedded within an anatomically distributed respiratory motor pattern generating network, we locally disinhibited the nucleus of the solitary tract (NTS), the pre-Bötzinger complex/Bötzinger complex area (pre-BötC/BötC), the Kölliker-Fuse nuclei (KFn) or the periaqueductal gray (PAG)—brainstem and midbrain areas known to be involved in the generation or modulation of the respiratory rhythm and pattern—by microinjecting the GABA(A)R antagonist bicuculline (50 nl, 10 mM). **(A–D)** Representative traces are shown at baseline and after local disinhibition of the NTS **(A)**, pre-BötC/BötC **(B)**, KFn **(C)**, or PAG **(D)**. The eupneic three-phase motor pattern observed at baseline was disrupted by local disinhibition of any brainstem target site **(A–C)**. In contrast, local disinhibition of the PAG evoked a modulation of the respiratory motor pattern that spared the eupneic three-phase motor pattern **(D)**. The PAG disinhibition-evoked modulation of the respiratory pattern consisted of stationary eupneic breathing intermingled with tachypneic events that were reminiscent of physiologic modulations of the respiratory pattern like sniffing. PNA: phrenic nerve activity; VNA: vagal nerve activity. *Scale bars: 10 s.*

Because local disinhibition often disrupted the three-phase respiratory rhythm (see Figure 2), we developed a quantitative description of the respiratory pattern. We reasoned that the eupneic three-phase respiratory motor pattern could be quantified by the strength of synchronization between the coupled oscillatory respiratory motor outputs (e.g., PNA and VNA).

Because the activity of the phrenic and vagal nerves span the majority of the respiratory cycle, and because their signal-to-noise ratios permit the estimation of their instantaneous phases via the Hilbert transform method (Kralemann et al., 2008; Zhu et al., 2013), we chose to use the phase synchronization between PNA and VNA to define the respiratory motor pattern at baseline and after local disinhibition of a target site.

In essence, this approach allows us to model the respiratory motor activities under consideration as a system of coupled oscillators. We first confirmed that their activity was periodic by computing their power spectral densities using the Welch method. Next, we band-pass filtered the integrated signals around the respiratory frequency. Instantaneous protophases were then extracted by applying the Hilbert transform. Instantaneous phases were then determined by applying the transformation defined in Kralemann et al. (2008).

To quantify the strength of the phase synchronization interaction between PNA and VNA and to determine the significance of the phase synchronization interaction, we computed the mutual information of the instantaneous phases (Zhu et al., 2013; Dhingra et al., 2017). Mutual information, a measure of the statistical dependence between two variables, was computed from the joint-probability histogram according to the following equation:

$$\begin{array}{l} I\left(\varphi_{PNA};\varphi_{VNA}\right)\;=\;-\int_{0}^{2\pi }\;\int_{0}^{2\mathrm{03C0}}\;P\left(\varphi_{PNA},\varphi_{VNA}\right).\\ \ln\frac{P\left(\varphi_{PNA},\varphi_{VNA}\right)}{P\left(\varphi_{PNA}\right)P\left(\varphi_{VNA}\right)}d\varphi_{PNA}d\varphi_{VNA} \end{array}$$

where *P*(*φ*_PNA_, *φ*_VNA_) is the joint probability distribution of the instantaneous phases, and *P*(*φ*_PNA_) and *P*(*φ*_VNA_) are the marginal probability distributions of either variable. For all measurements of mutual information of the instantaneous phases, we used a fixed bin width of 0.03 rad to discretize the probability distributions. Because we use the natural logarithm in computing mutual information, reported mutual information values are presented in the corresponding unit of nats. Values of mutual information near zero indicate that the variables are independent and have no coupling, whereas high values of mutual information are associated with high dependence between the instantaneous phase variables, and thereby associated with a high synchronization strength between the pair of motor outputs.

To assess whether a given trial had a statistically significant interaction between the input and the output, we bootstrapped the distribution to represent the null hypothesis that the two instantaneous phases were independent by randomly shuffling the inter-event intervals and re-computing the instantaneous phases and their mutual information. The bootstrapping procedure was iterated 100 times to estimate the distribution that represented the null hypothesis. If a forcing trial had mutual information greater than the 99% confidence interval of the bootstrap distribution, it was considered significant.

All analyses were performed using custom routines implemented in MATLAB. Statistical analyses were performed in R. All measurements are reported as the mean ± standard deviation. Unless stated otherwise, statistical comparisons were made by applying a one-way or two-way ANOVA. The Tukey HSD *post hoc* test was used to identify specific differences.

## Results

To investigate the distributed organization of the brainstem network regulating the respiratory motor pattern, we locally disinhibited the nucleus of the solitary tract (NTS), pre-Bötzinger complex/Bötzinger complex area (pre-BötC/BötC), the Kölliker-Fuse nuclei (KFn), or the periaqueductal gray (PAG). Representative traces before and after local disinhibition are shown in Figure 2. At baseline, all preparations generated a sequential eupneic three-phase respiratory motor pattern that consisted of inspiration, post-inspiration, and late-expiration (Figures 2A–D, left panels). Eupneic phrenic nerve activity (PNA) began abruptly at the onset of inspiration and increased in amplitude in a ramp-like fashion until the onset of post-inspiration. The eupneic pattern of vagal nerve activity (VNA) began at the onset of inspiration and increased logarithmically until the onset of post-inspiration. The onset of post-inspiration was denoted by a peak in vagal nerve discharge that decayed exponentially until the onset of late-expiration, at which point, VNA ceased until the beginning of the next inspiratory phase.

Local disinhibition of the NTS, pre-BötC/BötC or KFn, but not the PAG, disrupted the sequential eupneic three-phase respiratory motor pattern (Figures 2A–D, *right panels*). Local disinhibition of the NTS enhanced VNA bursting but disrupted the coordination of PNA with VNA. Local disinhibition of the pre-BötC/BötC enhanced the frequency of PNA and VNA but also disrupted their eupneic coordination. Local disinhibition of the KFn enhanced VNA bursting, modulated the duration of inspiration, but did not change the frequency of the respiratory rhythm. Interestingly, despite the enhancement of VNA, local disinhibition of the KFn resulted in a pattern of PNA that alternated between short- and long-bursts. In contrast, local disinhibition of the PAG perturbed the respiratory motor pattern but remained eupneic. PAG-disinhibited respiratory motor patterns also contained brief tachypneic perturbations that maintained a three-phase respiratory motor pattern. Compared qualitatively to the motor pattern disruptions evoked by local disinhibition of ponto-medullary target sites (compare Figure 2D with Figures 2A–C), local disinhibition of the PAG appeared to evoke physiologic modulations of the respiratory motor pattern, akin to sniffing, rather than evoking the ataxic respiratory patterns observed after local disinhibition of brainstem respiratory areas.

Initially, to assess the effect of local disinhibition of a target site on the respiratory rhythm, we measured the respiratory phase durations and their variability using PNA as an index of the respiratory rhythm. Note that local disinhibition of brainstem-, but not midbrain-, respiratory areas disrupted the respiratory pattern to an extent such that the definition of the three-phase rhythm no longer applied. As such, the following analysis should be interpreted to reflect properties of the respiratory rhythm as expressed in PNA.

**Figure 3:**
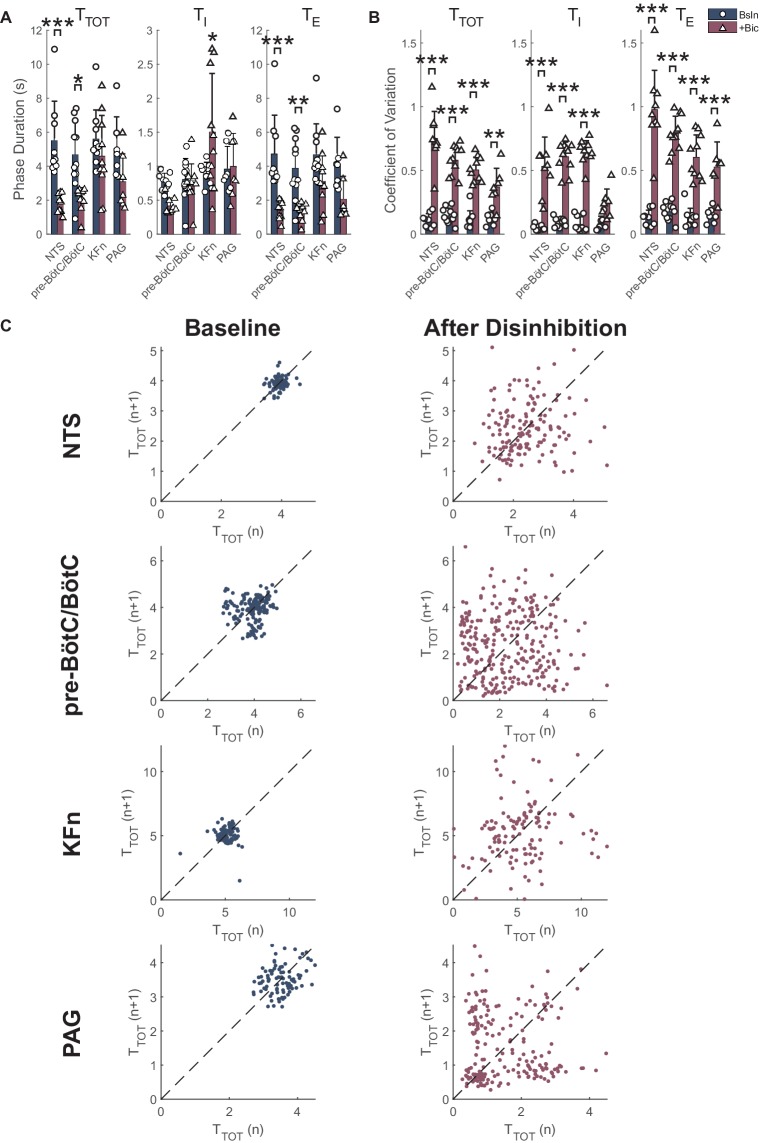
Local disinhibition of brainstem respiratory areas increases stochastic respiratory rhythm variability, whereas local disinhibition of the midbrain PAG increases deterministic respiratory rhythm variability. **(A)** The mean respiratory period (*T*_TOT_), inspiratory (*T*_I_), and expiratory (*T*_E_) durations were measured from PNA at baseline (*navy-colored bars*) and after local disinhibition (*wine-colored bars*) of the NTS, pre-BötC/BötC, KFn or PAG. Note that due to the disruption of the three-phase motor pattern evoked by disinhibition of brainstem respiratory target areas, these data should be interpreted to reflect the properties of the respiratory rhythm as expressed in PNA. Local disinhibition of the NTS significantly reduced the respiratory period due to a reduction in expiratory duration. Similarly, local disinhibition of the pre-BötC/BötC significantly decreased the respiratory period, and expiratory phase duration. Finally, local disinhibition of the KFn significantly increased the duration of inspiration but did not change the respiratory period. **(B)** Local disinhibition of any target site evoked a significant increase in the coefficient of variation FIGURE 3(CV) of the respiratory period and expiratory phase durations. The CV of *T*_I_ was significantly increased after local disinhibition of the NTS, pre-BötC/BötC and KFn, and tended to increase after local disinhibition of the PAG. **(C)** Representative Poincaré plots of the instantaneous respiratory period (*T*_TOT_) are shown at baseline (*left*) and after local disinhibition (*right*) of the NTS, pre-BötC/BötC, KFn or PAG. At baseline, all preparations had limited variability that appeared stochastic since the point clouds were clustered around the line of identity (*dashed lines*). After local disinhibition of the NTS, pre-BötC/BötC or KFn, the variability of the respiratory rhythm (width of the point cloud) increased, but still appeared stochastic. In contrast, after local disinhibition of the PAG, the tachypneic events that occurred regularly (see Figure 2D) were reflected by additional point clouds offset from the line of identity. This observation suggests that increasing the excitability of the PAG introduced a deterministic source of respiratory rhythm variability. ^*^p < 0.05; ^**^p < 0.01; ^***^p < 0.001.

Group data of the effect of local disinhibition on respiratory phase durations are presented in Figure 3A. Local disinhibition of the NTS significantly reduced the respiratory period (*T*_TOT_, from 5.5 ± 2.3 to 1.8 ± 0.6 s, *p < 0.001*, *N* = 9), and the duration of expiration (*T*_E_, from 4.7 ± 2.3 to 1.4 ± 0.5 s, *p < 0.001*, *N* = 9), but not the duration of inspiration (*T*_I_). Local disinhibition of the pre-BötC/BötC significantly reduced *T*_TOT_ (from 4.7 ± 2.0 to 2.1 ± 0.7 s, *p < 0.05*, *N* = 11), and *T*_E_ (from 3.9 ± 1.8 to 1.3 ± 0.5 s, *p < 0.01*, *N* = 11), but not *T*_I_. Local disinhibition of the KFn did not change *T*_TOT_ or *T*_E_, but significantly increased *T*_I_ (from 0.9 ± 0.2 to 1.5 ± 0.9 s, *p < 0.05*, *N* = 10). Finally, local disinhibition of the PAG tended to decrease *T*_TOT_ (from 5.0 ± 1.9 to 3.1 ± 1.7 s, *N* = 7) via a reduction in *T*_E_, but neither change was significant for the group. Together, these data show that local disinhibition of the NTS, pre-BötC/BötC, or KFn had a greater influence on the respiratory rhythm compared to local disinhibition of the PAG.

Group data of the effect of local disinhibition on the variability of the PNA rhythm are shown in Figure 3B. Local disinhibition of the NTS significantly increased the coefficient of variation of *T*_TOT_ [CV(*T*_TOT_) (from 0.11 ± 0.05 to 0.75 ± 0.21, *p < 0.001*, *N* = 9), CV(*T*_I_) (from 0.05 ± 0.02 to 0.53 ± 0.23, *p < 0.001*, *N* = 9), and CV(*T*_E_) (from 0.13 ± 0.06 to 0.98 ± 0.31, *p < 0.001*, *N* = 9)]. Similarly, local disinhibition of the pre-BötC/BötC significantly increased CV(*T*_TOT_) (from 0.14 ± 0.06 to 0.58 ± 0.11, *p < 0.001*, *N* = 11), CV(*T*_I_) (from 0.09 ± 0.03 to 0.61 ± 0.12, *p < 0.001*, *N* = 11) and CV(*T*_E_) (from 0.17 ± 0.07 to 0.79 ± 0.14, *p < 0.001*, *N* = 11). Local disinhibition of the KFn significantly increased CV(*T*_TOT_) (from 0.11 ± 0.07 to 0.50 ± 0.10, *p < 0.001*, *N* = 10), CV(*T*_I_) (from 0.08 ± 0.06 to 0.64 ± 0.09, *p < 0.001*, *N* = 10) and CV(*T*_E_) (from 0.13 ± 0.08 to 0.60 ± 0.18, *p < 0.001*, *N* = 10). Finally, local disinhibition of the PAG significantly increased CV(*T*_TOT_) (from 0.12 ± 0.05 to 0.37 ± 0.15, *p* < 0.01, *N* = 7) and CV(*T*_E_) (from 0.15 ± 0.06 to 0.53 ± 0.20, *p* < 0.001, *N* = 7), but not CV(*T*_I_). Together, these data suggest that local disinhibition of any target site increases the variability of the respiratory rhythm and expiratory intervals. However, whereas local disinhibition of brainstem respiratory areas increased the variability of inspiration, local disinhibition of the PAG did not modulate the variability of inspiration.

Representative Poincaré plots of the instantaneous respiratory period (*T*_TOT_) highlight the qualitative difference in the respiratory rhythm variability evoked by local disinhibition of brainstem respiratory areas versus the PAG (Figure 3C). At baseline, all preparations had a consistent respiratory rhythm with limited stochastic variability which was centered around the line-of-identity. In some cases, like the baseline condition before pre-BötC/BötC and PAG disinhibition, the variability of the respiratory rhythm in the perfused preparation can show some deterministic structure that may indicate the presence of consistent breath-to-breath sequences of respiratory cycle durations in the underlying time series. After local disinhibition of the NTS, pre-BötC/BötC, or KFn, all preparations showed a marked increase in the variability of *T*_TOT_ that was reflected by an increase in the size of the point cloud. The variability of the respiratory rhythm evoked by local disinhibition of any of these brainstem respiratory areas appeared random. In contrast, local disinhibition of the PAG was associated with a modulation of the respiratory pattern such that brief (two to four cycles) tachypneic events were intermingled with stationary breathing. In the Poincaré plot after local disinhibition of the PAG, this structured variability was reflected by the presence of clouds of points offset from the line of identity. This qualitative analysis suggests that local disinhibition of brainstem-respiratory areas increased the stochastic variability of the respiratory rhythm, whereas local disinhibition of the PAG introduced a deterministic source of variability into the respiratory rhythm.

**Figure 4:**
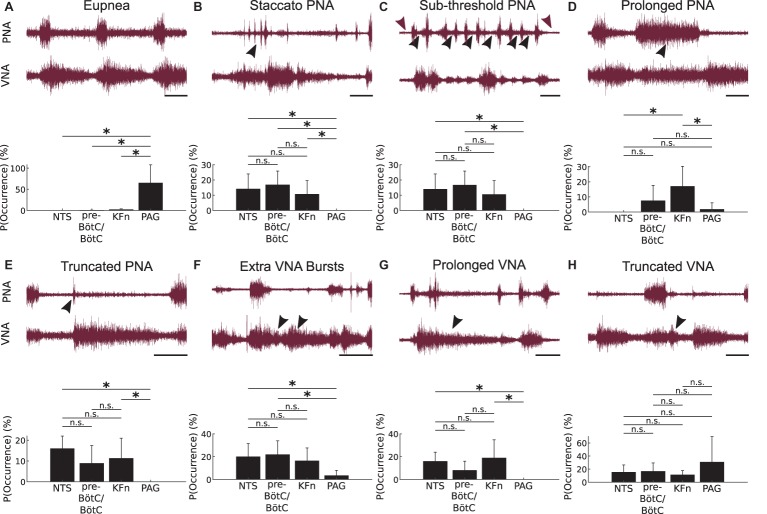
Local disinhibition of brainstem-, but not midbrain-, respiratory areas evokes ataxic respiratory motor patterns composed of a similar set of motifs. To qualitatively analyze the ataxic respiratory motor patterns expressed after local disinhibition of the NTS, pre-BötC/BötC, KFn or PAG, we scored the occurrence of eight representative respiratory motor pattern motifs during 3-min epochs recorded after bicuculline microinjection. The representative traces shown in this figure depict the definitions of the motor pattern motifs scored after local disinhibition of each target compartment. Below each set of raw traces, we plot the probability (or relative frequency) of observing a given respiratory motor pattern motif after local disinhibition of each target respiratory area. **(A)** *Eupnea*: The eupneic motif is defined by inspiratory activity on PNA, and by a bi-phasic VNA discharge that begins during inspiration, peaks at the onset of post-inspiration, and decays abruptly at the onset of the late-expiratory phase. As noted in Figure 2, the eupneic respiratory motor pattern motif was disrupted after local disinhibition of brainstem respiratory areas, but was spared after local disinhibition of the midbrain PAG. Further, the probability of observing eupneic fictive breaths was significantly greater after local disinhibition of the PAG compared to local disinhibition of either of the three brainstem respiratory areas. **(B)** *Staccato PNA*: This motif was defined by repeated, short bursts of PNA (*arrowhead*) associated with inspiratory-like discharges on VNA. There was no significant difference between the probability of occurrence of staccato PNA motifs after local disinhibition of brainstem respiratory areas. However, this motif was observed significantly less frequently after local disinhibition of the PAG. **(C)** *Sub-threshold PNA*: This motif was defined by the sub-threshold tonic discharge on PNA (*sub-threshold activity indicated by black- versus DC-level of noise indicated by wine-colored arrowheads, respectively*). This motif occurred significantly less frequently after PAG-disinhibition compared to disinhibition of brainstem respiratory areas. **(D)** *Prolonged PNA*: This motif was defined by apneustic bursts (*arrowhead*) reminiscent of those evoked by KFn lesions. This apneustic motif occurred significantly more frequently after KFn-disinhibition compared to after NTS-, or PAG-disinhibition. There was no significant difference in the probability of prolonged PNA motif occurrence after NTS-, pre-BötC/BötC- or PAG-disinhibition. **(E)** *Truncated PNA*: In this motif, we observed a short burst of PNA (*arrowhead*) that was quickly terminated by an exaggerated post-inspiratory discharge of VNA. This motif occurred significantly less frequently after PAG-disinhibition compared to after disinhibition of the NTS or KFn. Further, there was no significant difference in the probability of observing truncated PNA motifs after NTS-, pre-BötC/BötC- or KFn-disinhibition. **(F)** *Extra VNA Bursts*: This motif was characterized by the presence of one or more additional bursts or peaks of VNA discharge (*arrowheads*) after the initial VNA burst at the onset of post-inspiration. This motif occurred significantly less frequently after PAG-disinhibition compared to after NTS- or pre-BötC/BötC-disinhibition. There was no significant difference in the probability of observing extra VNA burst motifs after local disinhibition of brainstem respiratory areas. **(G)** *Prolonged VNA*: This motif was characterized by an exaggerated and sustained VNA (*arrowhead*) that continued through multiple cycles of PNA. This motif occurred significantly more frequently after NTS- or KFn-disinhibition compared to after PAG-disinhibition. There was no significant difference in the probability of observing this motif after local disinhibition of brainstem respiratory areas. **(H)** *Truncated VNA*: This motif was characterized by a rapidly decaying post-inspiratory discharge of VNA (*arrowhead*) that was very short compared to baseline post-inspiratory VNA. There was no significant difference in the probability of occurrence of this motif after local disinhibition of any respiratory area. *Scale bars: 2 s; n.s.: not significant;* ^*^p < 0.05.

To further analyze the motor patterns expressed after local disinhibition of respiratory areas, we scored the occurrence of eight representative motor pattern motifs during a 3-min epoch after local disinhibition (Figure 4). Representative traces for each of these motor pattern motifs are shown in Figure 4. To compare motif occurrence after local disinhibition of the NTS, pre-BötC/BötC, KFn, or PAG, we normalized the motif counts in each epoch to the total number of events to determine the probability of motif occurrence within each epoch.

The eupnea motif (Figure 4A) is defined by inspiratory activity on PNA, and by a bi-phasic VNA discharge that begins during inspiration, peaks at the onset of post-inspiration, and decays exponentially until the onset of the late-expiratory phase. The eupnea motif was almost never observed after local disinhibition of brainstem target sites but was preserved after PAG disinhibition. For the group, the probability of eupnea motif occurrence after PAG disinhibition was significantly greater compared to local disinhibition of the NTS, pre-BötC/BötC, or KFn (*p < 0.05*).

The staccato PNA motif (Figure 4B) was defined by repeated, short bursts of PNA associated with inspiratory-like discharges on VNA. The probability of staccato PNA motif occurrence was equally likely after NTS-, pre-BötC/BötC-, or KFn-disinhibition. After local disinhibition of the PAG, this motif never occurred and was significantly less likely to occur compared to local disinhibition of brainstem areas (*p < 0.05*).

The sub-threshold PNA motif was defined by the sub-threshold tonic discharge on PNA (compare black- versus wine-colored arrowheads in Figure 4C). Note that in the representative example shown, the tonic sub-threshold PNA was also associated with additional full amplitude PNA bursts during the period of tonic discharge. Sub-threshold PNA motifs were observed after NTS-, pre-BötC/BötC-, or KFn-disinhibition but not after PAG-disinhibition. The probability of sub-threshold PNA motif occurrence was equally likely after local disinhibition of brainstem areas. The probability of sub-threshold PNA motif occurrence was significantly less after local disinhibition of the PAG compared to after local disinhibition of the NTS or pre-BötC/BötC (*p < 0.05*).

The prolonged PNA motif (Figure 4D) was defined by apneustic bursts reminiscent of those evoked by KFn lesions. Less severe apneustic PNA bursts were also observed after pre-BötC/BötC- or PAG-disinhibitions. For the group, the probability of prolonged PNA motif occurrence was equally likely after NTS-, pre-BötC/BötC-, or PAG-disinhibition. After local disinhibition of the KFn, the probability of prolonged PNA motif occurrence was significantly greater compared to after NTS- or PAG-disinhibition (*p < 0.05*).

In the truncated PNA motif (Figure 4E), we observed a burst of PNA that is quickly terminated by an exaggerated post-inspiratory discharge of VNA. This motif was observed after local disinhibition of the NTS, pre-BötC/BötC, or KFn, but not the PAG. The probability of truncated PNA motif occurrence was equally likely after NTS-, pre-BötC/BötC-, or KFn-disinhibition. The probability of this motif occurring was significantly less after local disinhibition of the PAG compared to local disinhibition of the NTS or KFn (*p < 0.05*).

The extra VNA bursts motif (Figure 4F) was characterized by the presence of one or more additional bursts or peaks of VNA discharge after the initial VNA burst at the onset of post-inspiration. This motif was observed after local disinhibition of any respiratory area. The probability of extra VNA burst occurrence was equally likely after local disinhibition of the NTS, pre-BötC/BötC, or KFn. After local disinhibition of the PAG, the probability of this motif occurring was significantly less compared to after local disinhibition of the NTS or pre-BötC/BötC (*p < 0.05*).

The prolonged VNA motif (Figure 4G) was characterized by an exaggerated and sustained VNA that continued through multiple cycles of PNA. This motif was observed after local disinhibition of the NTS, pre-BötC/BötC, or KFn but never after PAG disinhibition. The probability of prolonged VNA motif occurrence was equally likely after NTS-, pre-BötC/BötC-, or KFn-disinhibition. The probability of this motif occurring was significantly greater after NTS- or KFn-disinhibition compared to after PAG-disinhibition (*p < 0.05*).

Finally, the truncated VNA motif (Figure 4H) was characterized by a rapidly decaying post-inspiratory discharge of VNA that was very short compared to baseline post-inspiratory VNA. For the group, the probability of truncated VNA motif occurrence was equally likely after local disinhibition of any site.

In general, the distributions of motif occurrence probabilities after local disinhibition of the NTS, pre-BötC/BötC and KFn clustered together, whereas the set of motifs occurring after local disinhibition of the PAG was in most cases significantly different from brainstem respiratory areas. Taken together, this analysis suggests that local disinhibition of the NTS, pre-BötC/BötC or KFn areas evokes a convergent set of respiratory motor pattern motifs, especially when compared to the breathing patterns evoked by local disinhibition of the PAG.

Because the previous qualitative analyses were prone to variability due to their subjective nature (e.g., manual scoring of phase durations and motif occurrence), we next sought to apply a more quantitative method to compare the effect of disinhibition of the NTS, pre-BötC/BötC, KFn, or PAG on the respiratory motor pattern. We assumed that the deviations from the eupneic three-phase respiratory rhythm could be quantified via changes in the strength of synchronization between respiratory motor outputs. Because PNA and VNA remained periodic after local disinhibition of any target site, and because the instantaneous phases of PNA and VNA could be determined in an un-biased fashion using the Hilbert transform method, we measured the phase synchronization between PNA and VNA to quantitatively analyze the disruption of the respiratory motor pattern after local disinhibition of target sites.

**Figure 5:**
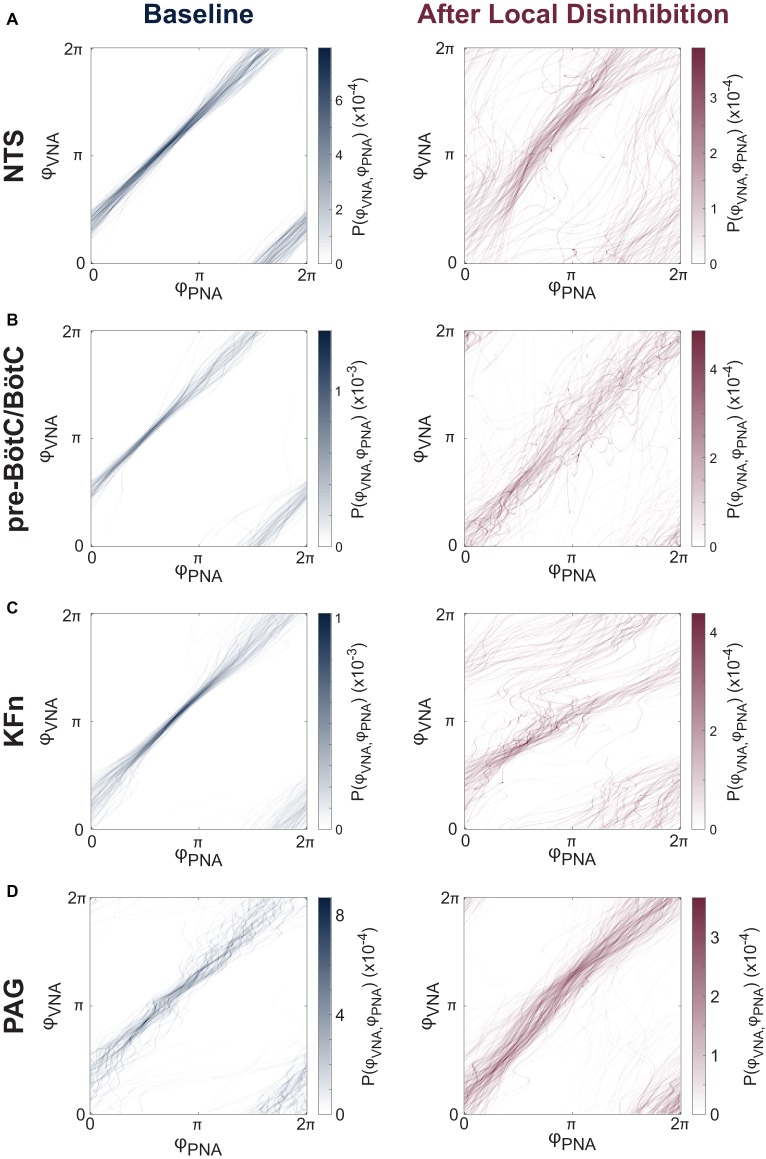
Local disinhibition of brainstem-, but not midbrain-, respiratory circuit compartments reduces the strength of PNA-VNA synchrony. **(A–D)** We assumed that the eupneic three-phase respiratory rhythm could be quantified via the synchrony between respiratory motor outputs. Because PNA and VNA remained periodic after local disinhibition of any target site, and because the instantaneous phases of PNA and VNA could be determined in an un-biased fashion by applying the Hilbert transform, we characterized the phase synchronization between PNA and VNA to quantitatively analyze the disruption of the respiratory motor pattern after local disinhibition of target sites. Representative joint probability distributions of the instantaneous phase of PNA (*φPNA*) and VNA (*φVNA*) are shown at baseline (*left*) and after local disinhibition (*right*) of the NTS **(A)**, pre-BötC/BötC **(B)**, KFn **(C)** or PAG **(D)**. The joint probability histograms are representative examples FIGURE 5from single experiments that reflect all respiratory cycles in a given epoch. Banding patterns in these histograms reflect the presence of a phase synchronization interaction between PNA and VNA. As expected, at baseline, all preparations showed strong phase synchronization between PNA and VNA. After local disinhibition of any brainstem target site **(A–C)**, many trajectories around this toroidal phase space deviated from the strongly synchronized trajectory observed at baseline. However, an interaction (banding pattern) remained between the instantaneous phases of PNA and VNA, suggesting that some component of the physiologic inspiration-to-post-inspiration transition remained after local disinhibition of any target compartment. In contrast, after local disinhibition of the PAG, the trajectories around the toroidal phase space remained clustered suggesting that local disinhibition of the PAG spared the eupneic phase synchronization interaction between PNA and VNA **(D)**.

Representative joint probability distributions of the instantaneous phases of PNA and VNA are shown before and after local disinhibition in Figure 5. In these plots, diagonal banding patterns appear when the instantaneous phases of PNA and VNA maintain a constant relative phase difference consistently for many cycles. Thus, the presence of such banding patterns indicates a potential phase synchronization interaction between PNA and VNA. As expected, at baseline (Figure 5, left panels), PNA and VNA were tightly synchronized. After local disinhibition of any target site (Figure 5, right panels), the non-eupneic respiratory motor pattern motifs were reflected as trajectories that deviated from the synchronized path around the torus. Nonetheless, for many respiratory cycles, PNA and VNA remained partially synchronized.

**Figure 6:**
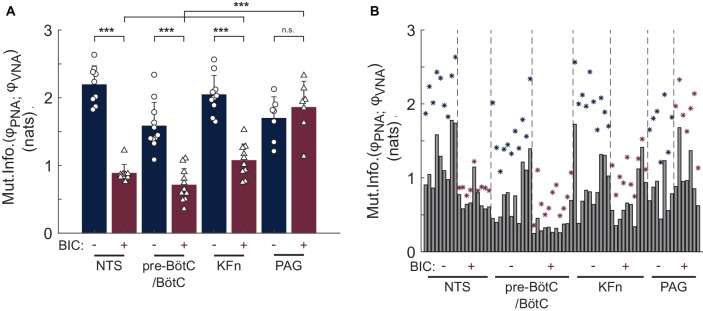
Group data reveal a quantitative difference in the effect of local disinhibition of brainstem-versus midbrain-respiratory areas on the strength of PNA-VNA synchrony. The mutual information of the instantaneous phases of PNA and VNA quantifies the strength of the phase synchronization between these two respiratory motor outputs. **(A)** After local disinhibition of any brainstem-, but not midbrain-, target site, the strength of the phase synchronization between PNA and VNA was significantly reduced. Further, the mutual information of the instantaneous phases of PNA and VNA after local disinhibition of the PAG was significantly greater than that after local disinhibition of the NTS, pre-BötC/BötC or KFn. **(B)** To assess the statistical significance of the phase synchronization interaction, we compared the mutual information of the original dataset with that of a surrogate distribution which was generated by shuffling whole cycles of the instantaneous phases. This plot shows the observed mutual information (*navy- or wine-colored stars*) and the upper-bound of the 99% confidence interval of the surrogate distributions (*gray bars*). Both before and after local disinhibition of target sites, the original mutual information of the instantaneous phases was greater than that of the respective surrogate distribution indicating the presence of a significant phase synchronization interaction between PNA and VNA. Taken together, the measurement of phase synchronization before and after local disinhibition of brainstem target sites suggests that a significant, but weaker, phase synchronization interaction persisted after local disinhibition of the NTS, pre-BötC/BötC or KFn. In contrast, the synchronization of PNA and VNA was preserved after local disinhibition of the PAG. ^***^p < 0.001; n.s.: not significant.

The mutual information of the instantaneous phases of PNA and VNA quantifies the strength of phase synchronization between these two respiratory motor outputs. Group data regarding the mutual information statistic are shown before and after local disinhibition in Figure 6A. For the group, local disinhibition of the NTS significantly reduced the mutual information of PNA and VNA (from 2.19 ± 0.28 to 0.88 ± 0.13 nats, *p < 0.001*, *N* = 9). Similarly, local disinhibition of the pre-BötC/BötC significantly reduced the mutual information of PNA and VNA (from 1.58 ± 0.35 to 0.71 ± 0.24 nats, *p < 0.001*, *N* = 11). Local disinhibition of the KFn also significantly reduced the mutual information of PNA and VNA (from 2.04 ± 0.29 to 1.07 ± 0.24 nats, *p < 0.001*, *N* = 10). Finally, local disinhibition of the PAG tended to increase the mutual information of PNA and VNA (from 1.69 ± 0.32 to 1.86 ± 0.39 nats, *N* = 7), but this change was not significant. Importantly, the mutual information of PNA and VNA after local disinhibition of the PAG was significantly greater than that evoked by local disinhibition of the NTS, pre-BötC/BötC, and KFn (*p < 0.001*).

To assess whether the presence of phase synchronization between PNA and VNA in each epoch was not simply due to chance, for each analyzed epoch, we generated a surrogate dataset by shuffling whole cycles of the instantaneous phases of PNA and VNA to determine the 99% confidence interval for the mutual information statistic. The original values of the mutual information of PNA and VNA (stars) and the upper-bound of the 99% confidence interval of the surrogate dataset (solid bars) are shown in Figure 6B. As expected, at baseline, all preparations showed a significant phase synchronization interaction between PNA and VNA, i.e., the original mutual information of the instantaneous phases was greater than the upper-bound of the 99% confidence interval of the surrogate dataset. Interestingly, after local disinhibition of the NTS, pre-BötC/BötC, KFn, the phase synchronization between PNA and VNA remained significant despite the change in the mutual information of the instantaneous phases. Taken together, these data demonstrate that a significant, but weaker, phase synchronization interaction persists after local disinhibition of the NTS, pre-BötC/BötC, or KFn.

## Discussion

The present study shows that local disinhibition of the NTS, pre-BötC/BötC, or KFn—respiratory nuclei which are distributed along the rostro-caudal and dorso-ventral axes of the ponto-medullary brainstem—evokes convergent disruptions of the respiratory motor pattern. In contrast, disinhibition of the midbrain PAG, which is known to potently modulate respiration, does not perturb the eupneic respiratory motor pattern. Our data support the conclusion that the circuit underlying respiratory pattern formation is anatomically distributed across the ponto-medullary brainstem, rather than being localized solely within a core circuit in the ventrolateral medulla.

### Local Disinhibition of Anatomically Distinct Nuclei in the Ponto-Medullary Brainstem, but Not Midbrain, Evokes Convergent Disruptions of the Respiratory Motor Pattern

The present study qualitatively and quantitatively demonstrates convergence between the highly disturbed respiratory motor patterns evoked by locally increasing excitability within either the NTS, the pre-BötC/BötC, or the KFn areas, especially when compared to the effect of local disinhibition of the midbrain PAG.

Locally increasing excitability of a respiratory area via pharmacologic disinhibition enables the functional measurement (via respiratory motor outputs) of the net connectivity of the target area with the remainder of the respiratory network. We postulate that if there are few connections from the target to the network, we would expect that the respiratory motor pattern would be minimally impacted by the disinhibition. If there is dense connectivity from the target area to the network, we would expect the respiratory pattern to be strongly modulated by the disinhibition. In addition, the effect of a local disinhibition may also depend on the pattern of connections from a target to the rest of the network. If projections from the target to the network influence many types of respiratory neurons (inspiratory, post-inspiratory, late-expiratory, phase spanning, etc.), we would expect the disinhibition to disrupt the respiratory pattern because these pathways would not be coherently activated by the disinhibition, i.e., we would introduce a strong source of stochastic variability in the network. Alternatively, if the projections from the target to the network are themselves coherent, e.g., neurons in the target area project to one or a few cooperative respiratory neuron types, we would expect that local disinhibition of the target would modulate the respiratory pattern since these synergistic projections would be coherently activated despite the noisy pharmacologic activation of the target.

In our results, all tested areas had a strong influence on the respiratory motor pattern, as predicted by previous work (Wasserman et al., 2002; Dutschmann and Herbert, 2006; Smith et al., 2007; Zhang et al., 2009; Bautista and Dutschmann, 2014b; Dhingra et al., 2017). However, the data also suggested a difference between local disinhibition of brainstem- versus midbrain-respiratory areas. Local disinhibition of brainstem respiratory areas (NTS, pre-BötC/BötC, or KFn) evoked ataxic respiratory motor patterns that could be described as respiratory motor pattern disruptions, whereas local disinhibition of the midbrain PAG evoked coherent modulations of the respiratory motor pattern that spared the eupneic three-phase respiratory motor pattern. We speculate that the latter was likely due to the presence of coherently organized projections from the PAG to select expiratory components of the respiratory network.

While previous studies have also used local disinhibition as a perturbation of physiologic respiratory network dynamics *in vivo* (Wasserman et al., 2002; Zhang et al., 2009; Bongianni et al., 2010; Janczewski et al., 2013), the most relevant study in the context of the present results is that of Marchenko et al. who microinjected a cocktail of GABAergic and glycinergic antagonists into the pre-BötC or BötC in the perfused preparation of rats (Marchenko et al., 2016). Consistent with the results of the present study, these authors observed a disruption of the respiratory pattern after local disinhibition of either the pre-BötC or BötC. Our results extend their findings by characterizing the specific disruptions of the respiratory motor pattern and by comparing the local disinhibition of the pre-BötC/BötC core circuit with the local disinhibition of other brainstem- and midbrain-respiratory nuclei.

Local microinjection of bicuculline into brainstem target areas triggered the emergence of generally similar sets of ataxic respiratory motor pattern motifs, especially when compared to local disinhibition of the PAG. While subtle differences in the expression of respiratory motor pattern motifs were observed, in most cases, we showed that probability of respiratory motor pattern motif occurrence was equivalent after local disinhibition of brainstem respiratory areas, whereas the respiratory pattern evoked by local disinhibition of the PAG consisted largely of eupneic breaths.

We further analyzed the effect of local disinhibition of respiratory areas with an unbiased quantitative approach based on measuring the phase synchronization between the motor outputs of the respiratory network. This analysis revealed that the strength of the synchronization between phrenic and vagal motor activities was significantly reduced after all three perturbations of brainstem respiratory areas, whereas local disinhibition of the PAG spared the phase synchronization interaction between phrenic and vagal motor pools. Importantly, local disinhibition and the resultant increased excitability of any brainstem target respiratory area resulted in a similar decrease in the synchronization strength between phrenic and vagal motor activities. If one specific brainstem area would have shown a significantly different reduction in the coupling strength compared to the other brainstem areas, it would have been considered to play a more significant role in respiratory pattern formation. However, since such a difference between the magnitudes of the reduction in phase synchronization strength after disinhibition of brainstem target sites was not observed, our data imply that the functional rCPG network is distributed across ventral and dorsal brainstem areas.

In contrast, we have shown that local disinhibition of the midbrain PAG evoked a qualitatively different modulation of the respiratory rhythm, but not pattern, compared to the local disinhibition of brainstem target nuclei. Neuronal cell columns within the PAG have long been known to project to various ponto-medullary respiratory nuclei to modulate respiration and are thought to relay and integrate limbic and cortical respiratory commands into respiratory brainstem circuits (Faull et al., 2019). Thus far, it has been shown that the respiratory effects of PAG stimulation depend on the activities of the parabrachial nuclei (located dorso-medially to the KFn) and the NTS (Huang et al., 2000; Hayward et al., 2004). While these projections overlap with the brainstem target nuclei considered in the present study, it is also clear that local disinhibition of the PAG did not evoke the ataxic respiratory motor pattern motifs observed after NTS-, pre-BötC/BötC-, or KFn-disinhibition. This observation suggests that the descending connectivity of the PAG may not be sufficient to enable the PAG to have direct control of respiratory pattern formation (Farmer et al., 2014).

### Excitation-Inhibition Balance Is a Mechanism Underlying Respiratory Pattern Formation

An important observation of the present study is that locally increasing excitability within a node of the distributed rCPG network was sufficient to disrupt respiratory pattern formation suggesting that the balance between excitation and inhibition (E-I balance) may serve as a mechanism that underlies the physiologic generation of the eupneic three-phase respiratory motor pattern. In cortical circuits, both during spontaneous and sensory-evoked activities, excitation is accompanied by a lagging inhibition (Borg-Graham et al., 1996; Wehr and Zador, 2003; Mariño et al., 2005; Haider et al., 2006). E-I balance is thought to keep cortical circuits in a state of criticality that may enhance information transfer (Shew and Plenz, 2013).

In general, inhibitory neurons have been shown to be intermingled with excitatory neurons in in the NTS, pre-BötC/BötC area, and KFn (Guthmann et al., 1998; Fong et al., 2005; Winter et al., 2009). In the present study, we observed that locally increasing the excitability of only one anatomically-distinct brainstem target area was sufficient to completely disrupt the eupneic three-phase respiratory motor pattern. Thus, the present results are in accordance with a role for E-I balance in determining the respiratory pattern.

Considering the bicuculline microinjections in the context of E-I balance also helps to understand the paradoxical appearance of apneustic respiratory motor pattern motifs, especially after local disinhibition of the KFn. Importantly, all three target areas within the ponto-medullary brainstem were previously shown to be key elements for the mediation of the inspiratory off-switch because local pharmacological inhibition (lesion) of either the NTS, BötC, or KFn reportedly triggers apneusis (prolonged PNA motif, see Figure 4D; Cohen and Shaw, 2004; Dutschmann and Herbert, 2006; Burke et al., 2010; Bautista and Dutschmann, 2014a; Dhingra et al., 2017). Apneusis is a pathological prolongation of inspiration, and typically reflects a delay in the inspiratory off-switch mechanisms. While the evidence that pharmacologic inhibition of the KFn triggers apneusis supports a hypothesis that increasing the excitability of the KFn should inhibit inspiration and enhance post-inspiratory motor outputs, in the present study, we observed that increasing the excitability of the KFn paradoxically also triggers apneusis. It is important to note, however, that the apneusis triggered by disinhibition of the KFn was qualitatively different from the previously reported apneusis triggered by KFn inhibition (Dutschmann and Herbert, 2006; Dhingra et al., 2017). Previous studies have shown that KFn- (or NTS- or BötC-) inhibition evokes a stationary apneustic respiratory motor pattern in which every respiratory cycle contains a prolonged inspiratory effort without the expression of post-inspiratory VNA. In contrast, in the present study, we observed a highly variable apneustic respiratory motor pattern after disinhibition of the KFn. More specifically, as can be observed in the raw traces shown in Figure 2, local disinhibition of the KFn did not cause every respiratory cycle to be apneustic. Instead, we observed both severely apneustic respiratory cycles associated with a prolonged inspiratory effort and no post-inspiratory VNA, and truncated PNA motifs wherein inspiratory motor output in PNA was quickly terminated by a prolonged VNA motif. A similarly variable pattern of PNA was previously reported after local disinhibition of the BötC with bicuculline in anesthetized rabbits (Bongianni et al., 2010). Therefore, the results of the present study suggest that a mixture of excitatory and inhibitory inputs (from either local or long-range projections) to the KFn determines the timing of the inspiratory off-switch and highlight E-I balance as a mechanism underlying eupneic respiratory pattern formation. Consequently, the perturbation of E-I balance in any given brainstem node of the pattern generating circuit was sufficient to perturb the global respiratory network dynamics underlying the formation of the respiratory pattern.

Pathological changes in E-I balance are also known to underlie pathologic breathing patterns in Rett syndrome. Initially, excitability-related synaptic dysfunction was identified functionally in the NTS, KFn, locus coeruleus, and ventrolateral medulla (Stettner et al., 2007; Medrihan et al., 2008; Taneja et al., 2009; Kline et al., 2010; Abdala et al., 2016). However, from these studies alone, it was difficult to determine the causal source of pathologic breathing patterns in Rett syndrome. The findings of the present study show that disruption of E-I balance within any of these brainstem areas could potentially underlie Rett syndrome pathology. Consistent with this interpretation, later Fos-expression studies in MeCP2-deficient mice demonstrated that forebrain circuits are subject to widespread hypo-excitability, whereas brainstem networks are hyperexcitable (Kron et al., 2012). While E-I imbalance in the brainstem respiratory network will trigger neurogenic breathing disorders, the findings of the present study also suggest that a specific pathologic motor pattern may not necessarily be predictive of the anatomic location of the E-I imbalance. Thus, the findings of the present study extend a growing body of literature supporting the hypothesis that eupneic respiratory pattern formation depends on E-I balance within brainstem respiratory circuits.

### Technical Considerations

Bicuculline is classified as a competitive GABA(A) receptor antagonist. However, the aim of the present study was not to investigate the role of GABAergic inhibition within localized brainstem or midbrain nuclei. Instead, we used bicuculline microinjections as a pharmacologic tool to locally increase neuronal excitability. Thus, bicuculline-related side-effects, including the blockade of calcium-activated potassium conductances or GABA reuptake (Olsen et al., 1976; Heyer et al., 1981; Johnson and Seutin, 1997; Johansson et al., 2001), do not interfere with our conclusions. First, blocking GABA reuptake should have a marginal effect on neuronal excitability when local GABA(A) receptors are also blocked. Second, the apamin-like activity of bicuculline that blocks calcium-dependent potassium conductances should also increase neuronal excitability. Thus, potential off-target effects of bicuculline should further enhance local hyperexcitability.

## Conclusions and Future Directions

While effects of the network perturbation following bicuculline-evoked increases in excitability on the generation of the respiratory pattern (Bautista and Dutschmann, 2014b; Abdala et al., 2016) or reflex mediation (Dutschmann and Herbert, 1998) were previously reported, this is the first publication that qualitatively and quantitatively compared such effects of a local disinhibition. The role of the KFn as a key area for the control of respiration has been appreciated for many years (see Marckwald, 1887). The interaction between the KFn and pre-BötC/BötC core circuit is also considered in many computational and conceptual models (Rybak et al., 2004; Smith et al., 2007, 2009; Mörschel and Dutschmann, 2009; Dutschmann and Dick, 2012; Ausborn et al., 2018). Consequently, the anatomical location of the rCPG is commonly depicted as continuous bilateral respiratory columns that stretch from the ventral caudal medulla to the dorsolateral pons (see Alheid et al., 2004; Alheid and McCrimmon, 2008). However, the role of the NTS and adjacent dorsal brain structures of the medulla oblongata, as a dorsal respiratory group and integral part of the rCPG, was just recently re-discovered in experiments using the perfused brainstem preparation (Bautista and Dutschmann, 2014b; Jones et al., 2016) and further supported by the findings of the present study.

Overall, the data of the present study strongly suggest that the rCPG cannot be associated with a compact and continuous cell column that only extends rostrocaudally and instead needs to also be functionally and anatomically extended to dorsal brainstem area(s). Therefore, future investigations exploring the dynamical mechanisms underlying respiratory control should look broadly across respiratory neuronal populations to unravel the fundamental neurophysiologic principles controlling the breath. Our observations also highlight the need to implement modern experimental tools, like population imaging and multi-electrode array recording, in preparations that maintain the functional and anatomic integrity of the rCPG to optimally capture brainstem-wide respiratory circuit dynamics.

## Ethics Statement

Experimental protocols were approved by and conducted with strict adherence to the guidelines established by the Animal Ethics Committee of The Florey Institute of Neuroscience and Mental Health, Melbourne, Australia.

## Author Contributions

MD and RD designed and conceived the study. RD, WF, and TB conducted the experiments. RD, WF, and MD analyzed the data. RD, WF, TD, RG, and MD interpreted the data and wrote the manuscript.

### Conflict of Interest Statement

The authors declare that the research was conducted in the absence of any commercial or financial relationships that could be construed as a potential conflict of interest.
